# Research on the impact of technology mergers and acquisitions on corporate performance: an empirical analysis based on China’s pharmaceutical industry

**DOI:** 10.3389/fpubh.2024.1419305

**Published:** 2024-08-09

**Authors:** Jialin Yang, Jiawen Li, Su Wang, Yuwen Chen

**Affiliations:** ^1^School of Business Administration, Shenyang Pharmaceutical University, Shenyang, China; ^2^Drug Regulatory Research Base of NMPA, Research Institute of Drug Regulatory Science, Shenyang Pharmaceutical University, Shenyang, China

**Keywords:** technology M&A, corporate performance, R&D, pharmaceutical industry, mediating effects

## Abstract

There is intense competition among pharmaceutical companies with the rapid growth of the global pharmaceutical industry. In recent years, China has continuously increased the reform of the medical system. Technology mergers and acquisitions (M&A) in China’s pharmaceutical industry have emerged in this complex policy and economic background. This paper conducts an empirical study from the dual perspectives of financial performance and innovation performance, based on unbalanced panel data of Chinese listed pharmaceutical firms from 2012 to 2022. The impact of technology M&A on firm performance is analyzed in terms of the heterogeneity of firm characteristics. Meanwhile, the relationship between R&D investment in technology M&A and firm performance is examined. The results show that technology M&A can promote the performance of pharmaceutical companies, and R&D investment has a mediating effect on the impact of technology M&A on corporate performance. Based on the above findings, this study enriches the relevant literature on technology M&A in the pharmaceutical industry, provides warnings and suggestions for pharmaceutical companies to improve corporate performance through technology M&A, and provides reference materials for future policy formulation.

## 1 Introduction

Competition among enterprises is intensifying in the context of global economic integration (1). Governments have intensified their scientific and technological innovation backing to gain a competitive edge. It is an important indicator that the innovation capacity of pharmaceutical companies can measure a country’s strength in science and technology innovation (2). The pharmaceutical market has opportunities and challenges when China is economically transforming and upgrading (3). Pharmaceutical companies can improve their innovation performance in two ways: internal research and development (R&D) and external mergers and acquisitions (M&A). It is relatively slow to depend on internal R&D to enhance technology because of limitations in technical resources and research and development professionals (4). Therefore, there is a growing tendency among pharmaceutical companies to pursue advanced pharmaceutical development technologies to acquire specialized technological resources from external sources and to use technology mergers and acquisitions to achieve leapfrog innovation (5).

In recent years, China has been increasing its efforts to reform the pharmaceutical system and to develop technological innovations in pharmaceutical enterprises (6). There have been mergers and acquisitions of companies in the pharmaceutical industry in China’s complex policy and economic background. It is increasingly recognized among pharmaceutical companies that they can gain access to advanced technologies and products by engaging in technological mergers and acquisitions, which allows them to establish an advantageous market position (7). However, China’s pharmaceutical industry is relatively dispersed regarding industrial structure. Although the number of pharmaceutical enterprises is large, the scale is generally small, and the innovation ability is relatively weak. There is an apparent gap between Chinese pharmaceutical companies and large multinational pharmaceutical companies in developed countries regarding research and development capabilities, innovation capabilities, financial strength, etc. (8). China has implemented many measures to promote and facilitate the technological advancement of pharmaceutical companies in recent years. As an illustration, the government will offer tax advantages, financial assistance, and other policy measures to stimulate firms to enhance their investment in research and development. These policies create a favorable external environment and conditions for Chinese pharmaceutical businesses to engage in technology mergers and acquisitions, making such mergers and acquisitions an essential means to boost the development of the pharmaceutical industry.

The earliest concept of technology mergers and acquisitions (M&A) is Williamson’s 1975 proposal that technology M&A is a mergers and acquisitions activity with the primary goal of acquiring the target’s technological resources (9). Technology M&A is a highly effective technique for companies to quickly get innovative resources and strengthen their ability to innovate technologically in response to changes in their business models (10). Mergers and acquisitions among enterprises originated in the United States and have since expanded worldwide (11). Scholars from various countries have researched technology mergers and acquisitions, with the most influential researchers appearing in Sweden and the United States. Jacobsson and Granstrand revealed in 1983 and 1984, respectively, that small firms in technology M&As have seller characteristics in deal offers in the M&A market due to their advanced technology patents. Granstrand (12) discussed the role of technology M&A in the mergers and acquisitions process using the “theory of technology-based enterprises.” Wang and Han (13) examined how the absorptive capacity of American companies might influence technological innovation as a moderator. Enterprises’ absorptive capacity enables them to incorporate and utilize external technological information as internal knowledge effectively. It is a relatively late research on technology mergers and acquisitions in China. Wu et al. (14) examined the developmental trajectory of China’s firms’ technological prowess, as demonstrated through their adoption of new technologies, expansion of production capacity, and ability to innovate. The primary purpose of leading companies engaging in M&A is to address their deficiencies in specific areas, enhance the diversified growth of their technology research and development, and lay the foundation for comprehensive technological innovation in the future. Based on existing research on technology M&A, one view is that technology mergers and acquisitions can efficiently address R&D disadvantages and enhance the knowledge capacities of acquiring organizations (15). Simultaneously, technology M&A provides exit channels other than IPOs for the founders of target firms, thus reducing their entrepreneurial risk (16). This initiative aims to incentivize target firms to enhance their investment in research and development and intensify their efforts in technological knowledge innovation (7). Furthermore, Ghosh et al. (8) propose that technology M&A offers a faster way to obtain external technological resources than internal research and development. It is an effective way for enterprises to master the technical knowledge of the target enterprise and absorb high-tech talents. Another view is that technology M&A may hinder the improvement of internal research and development capabilities if firms rely too much on external technological resources. The enterprise’s intangible resources are not effectively accumulated, and the absorption of external knowledge resources will be negatively affected (17). Szucs (18) argues it will diminish the enterprise’s ability to innovate independently if the primary objective of technological mergers and acquisitions is to evade market competition rather than efficiently incorporate and utilize the acquired technology.

To sum up, the extant literature on technological M&A and enterprise performance lacks consensus and is confined to a singular perspective. Only a few numbers of researchers have empirically investigated technological M&A in the pharmaceutical industry. Different firms are affected differently by undertaking technology M&As. It is essential to consider the diversity of innovation subjects, whose characteristics such as property rights, size, and geographical location should also not be ignored. Therefore, it is important to further investigate the effect of technology M&A on firm performance and understand the mechanisms by which technology M&A enhances firm performance. This paper empirically analyses the impact of technology M&A on firm performance using a fixed-effects approach based on unbalanced panel data of listed pharmaceutical firms in China from 2012 to 2022. In addition, it comprehensively examines the diversity among enterprises with varying ownership characteristics, sizes, and geographic locations. It then further analyses the role of R&D investment as a mediator between technology M&A and firm performance. It offers rich materials for China’s pharmaceutical industry to launch technology M&As, aiding firms in gaining a deeper understanding of technological expansion through M&A to enhance their innovation capabilities. Furthermore, this article analyzes the outcomes of the present government’s strategy to encourage technological advancement in China and offers pertinent information for future policy development using the most recent sample data.

Compared with the existing results, this research offers possible contributions as follows: Firstly, it is the inaugural empirical study that examines financial performance and innovation performance from a dual perspective. The extensive literature on company performance mostly concentrates on individual performance indicators. This paper integrates the two variables into a single variable for research and analysis to systematically evaluate the influence of technological mergers and acquisitions on the performance of pharmaceutical manufacturing companies. It complements the limited theory and empirical evidence available in this field. Secondly, this paper explores the relationship between technology M&A and firm performance and frames the study of R&D inputs to explore its mechanism as a mediating variable. Existing literature has conducted a great deal of research and studies from the perspective of the respective impact of R&D inputs and technology M&A on firm performance, and some scholars have made several studies from the relevant aspects of individual firms. This paper will provide R&D inputs into the study of technology M&A and its impact on company performance, enriching the relevant literature. Thirdly, there are fewer studies on technology M&A in China’s pharmaceutical companies. This paper specifically studies the impact of technology M&A on firm performance in the pharmaceutical industry with Chinese characteristics (heterogeneity) to provide a realistic reference for the current innovation practice of pharmaceutical enterprises.

The rest of the paper is organized as follows. In the “Literature Review and Research Hypotheses” section, this paper reviews the previous literature and puts forward the hypotheses of this paper. The “Research Design” section provides data sources, variable selection, and model setup. The “Empirical Analysis” and “Heterogeneity Analysis” sections discuss the results. Finally, “Conclusions and Recommendations, Shortcomings and Prospects” is given.

## 2 Literature review and research hypothesis

### 2.1 Technology mergers and acquisitions and firm performance

With the growing requirement for enterprise innovation, obtaining external technical resources has become a major incentive for companies to combine and acquire. In theory, the mechanism by which companies rely on acquisitions to enhance innovation output is mainly reflected in two aspects. One is the selection mechanism; it is more efficient for companies with poor innovation capabilities to acquire innovation by acquiring companies with substantial expertise or ready-made patents than a direct investment in independent innovation (19). Cassiman et al. (20) argues that the principal merger party will purposefully select target firms that possess their missing technological knowledge so that they can update their existing knowledge after the technology acquisition. The study conducted by Chen (21) shows that there is a very important role in the development of new ideas and the existing knowledge base of firms in enhancing innovation core competitiveness after M&A. The second is synergy; it will be enlarged to cover the knowledge stock of the leading merging company after a technology M&A. It is beneficial for enterprises with a deep stock of knowledge to absorb external technological resources, which enhances their innovation capacity and increases the firm’s innovation output (22). Zhang et al. (23) showed that technology M&A can avoid the knowledge cocoon trap and innovation path dependence generated by long-term independent research and development, which can rapidly update and expand the existing knowledge stock of enterprises. Enterprises have complementary technological resources to enhance innovation power because of the synergistic effect.

Technology mergers and acquisitions are an effective strategy for enterprises to acquire innovative resources and enhance corporate performance rapidly. According to Zhao (24), an analysis of M&A cases across various industries in the United States between 1984 and 1997 revealed that M&A transactions driven by the goal of technology innovation are a common phenomenon. It is through technology M&As that companies, especially those with a weak innovation capacity before the M&A, will increase the number of patents obtained. Entezarkheir and Moshiri (25) argues that M&A significantly positively affects corporate innovation, and heterogeneity will exist across industries. Chinese scholars have shown that technology mergers and acquisitions clearly influence firms’ innovation performance (26). Qu (27) analyzed the intrinsic link between a company’s technology M&A and innovation performance and found that complementary and substitutive technology M&As significantly boost the firm’s innovation performance. Also, the study conducted by Yang and Zhou (28) demonstrated that the impact of technical innovation resulting from technology M&As becomes more evident when the acquired firm experiences significant growth. Wu et al. (29) investigated the effect of knowledge integration on firms’ innovation performance based on different technology M&A modes. Enterprises will change their knowledge base regarding width and depth when they carry out two modes of technology M&A. Therefore, the performance of innovation in enterprises will yield varying outcomes.

Additionally, Nesta and Saviotti (30) conducted an empirical study on the pharmaceutical industry and concluded that the higher the acquired firm’s technological R&D base, the more effective it is in improving the R&D capability of the primary acquiring firm after the merger. Also, it is more conducive for firms with a solid long-term technological R&D base to enhance post-merger innovation performance. Lin and Jang (31) examined merger and acquisition data from the United States pharmaceutical industry and argued that complementarities between firms can improve technological development and innovation. Firms should find companies in the same industry as theirs that match their size and technology for strategic integration. Hao and Ren (32) studied the evolution of issues related to technology M&A in high-tech enterprises. They proposed that the impact of technology M&A on the technological integration of enterprises varies depending on the industry. Technology M&A particularly promotes R&D in the pharmaceutical industry and suggests relevant countermeasures for enterprises and authorities. Yu and Wang (33) used a double-difference method to compare the innovation performance of technology M&As between firms that executed M&As and firms that did not perform M&As throughout different policy stages. The study results showed that firms engaging in technology M&As could improve their innovation performance in the short run before implementing the policy. Still, the innovation effect was negative in the long run. After implementing the policy, firms that engage in technology M&As show adverse innovation effects in the short term. The study results provide a realistic reference for the future decision-making of enterprises and the establishment of national policies.

Based on the combination of the above theories and literature, this paper proposes the following research hypotheses:

*H1*: Technology mergers and acquisitions positively affect firm performance.

### 2.2 The mediating role of R&D investment

There are two basic approaches to innovation for enterprises: closed innovation and open innovation. Closed innovation is mainly based on internal R&D, and R&D investment is the core bloodline of firms’ innovation activities (22), and firm performance is closely related. It can enhance the enterprise’s independent R&D capability, which starts from within the enterprise to invest enterprise resources in R&D activities. Enterprises can master the core technology and form their core competitiveness through independent R&D to occupy a favorable position in the market (34). One of the most important avenues for open innovation is technology M&A, which allows for rapid access to external technological resources and core knowledge capabilities and improves the firm’s innovation ability (35). Compared with the long time, high risk, and high investment required for independent R&D, technology M&A can more rapidly acquire the technological knowledge the target firm holds (4). Firms relying solely on internal R&D to realize innovation can increase riskiness in the context of the increasing speed of innovation iteration (36). There is a growing realization in enterprises that innovation does not only come from within the enterprise; external resource integration is also an essential part (37).

Williamson’s Transaction cost theory (9) states that external technological resource acquisition replaces internal R&D skills. Companies experience transaction costs when they acquire external technology resources, which impact the incorporation of these resources within the company. Cohen and Levinthal (6) contend that the firm’s internal R&D capacity plays a significant role in assimilating and innovating external resources. However, it will negatively impact the company’s performance due to an excessive dependence on external technology resources and a lack of logical incorporation of externally acquired technological resources. Hitt et al. (38) and Jensen (39) propose that the reduction in R&D spending by innovative companies following mergers and acquisitions diminishes their level of R&D intensity. Firms interrupt their existing development plans to use the target company’s resources better and spend a lot of time on strategic adjustments at the managerial level, slowing down technological innovation in the company. It has also been argued that inadequate integration measures are not taken after a merger or acquisition, or if there is inertia in autonomous innovation due to technology purchase, this can negatively impact a firm’s innovation performance (40). In addition, Wang and Ma (36) discovered that the R&D expenditure of the dominant party involved in a merger and acquisition has a moderating effect on the process using a multiple regression model. This moderation promotes the combination of resources and collaborative innovation. Gandal and Scotchmer (41) highlighted that corporate governance issues influence the optimal selection of R&D investment by decision-makers, which subsequently impacts the efficiency of using external technological resources.

In summary, this paper argues that after technology mergers and acquisitions, adequate integration measures are taken on acquired technological resources by increasing R&D investment, which leads to a growth trend in firm performance. Based on this analysis, this paper proposes the hypothesis:

*H2*: R&D investment mediates the effect of technology mergers and acquisitions on firms’ innovation performance.

## 3 Materials and methods

### 3.1 Sample selection and data source

This paper selects the information on M&A events of Chinese A-share listed pharmaceutical companies from 2012 to 2022 as the research sample. Based on data availability and accuracy, this paper excludes ST and *ST, PT, and companies with missing relevant data. For multiple M&A events of the same company in the same year, the first M&A event in a year is selected. Finally, 1,418 unbalanced panel observations for 145 firms that meet the requirements are obtained. To eliminate the impact of outliers on the study, this paper uses Stata16.0 software to perform bilateral shrinkage of the relevant variables. The financial data of listed companies are mainly obtained from the China Stock Market and Accounting Research Database (CSMAR, https://data.csmar.com/) and Juchao Information Network^1^ to find the annual reports of enterprises. Patent data comes from the China Research Data Service Platform (CNRDS, https://www.cnrds.com/).

### 3.2 Variable selection and definition

#### 3.2.1 Explained variable

The paper studies the effects of technology mergers and acquisitions on enterprise performance by thoroughly analyzing innovation and financial performance variables. It refers to the study by Gu and Xie (42) which selects return on assets (ROA) as the metric to evaluate corporate financial performance. Patents are highly effective in measuring innovation performance, as they provide significant exclusivity and can explain the rise in performance output resulting from technological innovation. A greater quantity of patent applications typically signifies a higher degree of innovation performance exhibited by a company. Experts commonly assert that the quantity of patent applications provides a more accurate indication of the extent of innovation compared to the number of grants. This is due to the fact that patent approvals necessitate evaluation and payment of yearly fees, leading to greater unpredictability and volatility. Thus, this study uses the total number of patent applications increased by one as the logarithm of the innovation performance indicator for measurement.

#### 3.2.2 Explanatory variables

Technology mergers and acquisitions (Tma) is a dummy variable, assigned a value of 1 if a technology merger or acquisition occurs in an enterprise. Otherwise, it is assigned a value of 0. Technology mergers and acquisitions provide a direct means of accessing the technological resources of the target firm and achieving the substitution and complementation of production technology. This paper defines technology M&A according to Ahuja and Katila (43). Technology M&A refers to M&A events involving listed businesses that meet one of the following three criteria: (i) the announcement of the M&A by the businesses listed clearly states that the goal of the M&A is to get technology. (ii) The target company possesses patented technology within 5 years prior to the date of the M&A announcement. (iii) The listed companies are classified as high-tech enterprises. This study utilizes the CSMAR database to extract the 2012–2022 M&A information table of listed businesses. We next manually examine the relevant papers to ascertain if the M&A events fall under the category of technological M&A. After applying the aforementioned criteria, we obtained a total of 293 samples of M&A events that satisfy the specified conditions.

#### 3.2.3 Mediating variable

Research and development (RD) investment. To measure R&D investment, the empirical practice of Guo (44) measures the R&D intensity of enterprises by the proportion of R&D expenditure to operating revenue.

#### 3.2.4 Control variable

The magnitude of a company’s assets directly impacts its capacity to effectively incorporate post-merger technology. Information asymmetry can create market flaws that lead to financing limits for organizations. Companies with large levels of debt typically incur more risks, which in turn restrict their involvement in technological mergers and acquisitions. Companies that experience higher rates of growth in their operating revenue typically possess stronger skills for achieving growth. They are more likely to be preferred by the capital market in mergers and acquisitions. In this paper, we cite the works of Hui et al. (45), Wang and Huang (46), and Hong (47) to regulate the variables of firm size, asset-liability ratio, financing constraints, operating income growth rate, and total asset turnover. This is done to enhance the scientific rigor and dependability of the study by managing other potential confounding factors. See Table 1 for specific measurements.

**Table 1:** Variables and their definitions.

Variable type	Variable name	Variable symbol	Variable description
Explanatory variable	Financial performance	Roa	Net profit/total assets
	Innovation performance	Invia	Natural logarithm of the number of patents for inventions plus 1
Explanatory variable	Technology mergers and acquisitions	Tma	Dummy variable, 1 if technology acquisition, 0 otherwise
Mechanism variables	R&D investment	RD	R&D investment as a percentage of operating income (%)
Control variable	Company size	Size	Natural logarithm of total business assets
	Asset liability ratio	Lev	Total liabilities divided by total assets
	Revenue growth rate	Gro	(Amount of operating revenues for the current single quarter – Amount of operating revenues for the previous single quarter)/Amount of operating revenues for the previous single quarter
	Total asset turnover	AT	Operating income/total assets
	Financing constraints	SA	SA index indicates the degree of financing constraints

### 3.3 Model construction

#### 3.3.1 Fixed effects model

To test the impact of technology mergers and acquisitions on corporate performance, this paper refers to the research of Hou (48). It combines theoretical analyses and the design of research indicators to construct the following econometric model:


(1) $$Y_{\mathrm{it}}=\beta_{0}+\beta_{1}{\mathrm{Tma}}_{\mathrm{it}}+\sum \beta_{k}\;{\mathrm{controls}}_{\mathrm{it}}+{\mathrm{firm}}_{i}+{\mathrm{year}}_{t}+\varepsilon_{i,t}$$

Where i denotes individual firms, t denotes the year, and $Y_{\mathrm{it}}$ s dependent variables are financial performance (Roa) and innovation performance (Invia). The independent variables are technology mergers and acquisitions (Tma) and all other possible control variables (controls), respectively. firmi and ${\mathrm{year}}_{t}$ denote individual and time-fixed effects, respectively, and $\varepsilon_{i,t}$ is a random error term. Considering that the individual perspective of pharmaceutical enterprises is not affected by time (enterprise ownership, high-tech enterprise qualification) and the time perspective is not affected by individual changes in the enterprise (industrial structure, GDP growth, years of education of the provincial population, and the macroeconomic environment), and thus the empirical design includes the enterprise individual fixed effects and the year fixed effects, the constructed model (1) is a bidirectional fixed effects model.

#### 3.3.2 Mediating effect model

To explore the relationship between technology mergers and acquisitions, R&D investment, and enterprise performance, according to the three-step mediating effect model proposed by Wen et al. (49), this paper adds the mediating variable R&D investment (Rd) based on the above-fixed effect model (1), and constructs the model as follows:


 (2) $${\mathrm{RD}}_{\mathrm{it}}=\alpha 0+\alpha_{1}{\mathrm{Tma}}_{\mathrm{it}}+\sum \alpha_{k}\;{\mathrm{controls}}_{\mathrm{it}}+{\mathrm{firm}}_{i}+{\mathrm{year}}_{t}+\varepsilon_{i,t}$$

(3) $$Y_{\mathrm{it}}=\gamma_{0}+\gamma_{1}{\mathrm{Tma}}_{\mathrm{it}}+\gamma_{2}{\mathrm{RD}}_{\mathrm{it}}+\sum \gamma_{k}\;{\mathrm{controls}}_{\mathrm{it}}+{\mathrm{firm}}_{i}+{\mathrm{year}}_{t}+\epsilon_{i,t}$$

In Equation (2), $\;{\mathrm{RD}}_{\mathrm{it}}$ is the mediating variable R&D input, and the rest of the symbols have the same meaning as in Equation (1) above Equation (3), is based on Eq. (1), with the addition of the variable of R&D investment, which is used to test the effect of technology mergers and acquisitions and R&D investment on corporate performance.

## 4 Empirical analyses

### 4.1 Descriptive statistics analysis

The descriptive statistics of each variable are shown in Table 2. It can be seen that the return on assets (Roa) of enterprises ranges from −0.154 to 0.226, with an average value of 0.061, indicating that the financial status of enterprises varies greatly. The number of invention patent applications (Invia) ranges from 0 to 1.857, with an average of 0.893, indicating some variation in firms’ innovation performance and that most pharmaceutical firms have low innovation performance. For the explanatory variables, the mean value of Technology Mergers and Acquisitions (Tma) is 0.207, and the variance is 0.405. The range of Research and Development (RD) is 0.32 ~ 23.06, and the variance is 3.892, which is a significant difference, indicating a great difference in Research and Development (RD) among enterprises. There is a great fluctuation in R&D investment in each company and each year. The maximum value of the capital debt ratio (Lev) is 0.815, and the minimum value is 0.042, indicating that the debt capacity of listed companies is not uniform. The minimum value of enterprise growth (Gro) is -0.46, and the maximum value is 1.162, indicating that listed companies’ development ability and growth opportunities in China’s pharmaceutical manufacturing industry vary more significantly. The value of financing constraint (SA) ranges from -4.747 to-3.233, and the larger the value of SA, the larger the financing constraint. The average value of financing constraint is-3.887, which shows that enterprises generally face the dilemma of financing constraint.

**Table 2:** Descriptive statistics.

Variable	Obs	Mean	Std.Dev.	Min	Max
Roa	1,418	0.061	0.063	−0.154	0.226
Invia	1,418	0.893	0.486	0	1.857
Tma	1,418	0.207	0.405	0	1
RD	1,418	5.037	3.892	0.32	23.06
Size	1,418	9.633	0.404	8.825	10.696
Lev	1,418	0.324	0.181	0.042	0.815
Gro	1,418	0.139	0.285	−0.46	1.162
AT	1,418	0.538	0.233	0.125	1.303
SA	1,418	−3.887	0.263	−4.717	−3.233

### 4.2 Correlation analysis

The results of the Pearson correlation analysis between the variables are shown in Table 3. It can be seen that the correlation coefficient between technology mergers and acquisitions (Tma) and enterprise financial performance (Roa) is 0.351, and the coefficient with innovation performance (Invia) is 0.336; there is a significant positive correlation. This indicates that with the increase in technology mergers and acquisitions, enterprise performance will also improve, which initially verifies H1. Research and development investment (RD) also has a significant positive correlation with enterprise performance (Roa) and innovation performance (Invia), which initially verifies H2. The study results show that the variance inflation factor VI*F* value is less than 10, and the absolute value of correlation coefficients between the rest of the variables is less than 0.8, indicating that the variables passed the multiple covariance test.

**Table 3:** Correlation analysis.

Variable	Roa	Invia	Tma	RD	Lev	Size	Gro	AT	SA	VIF
Roa	1.000									
Invia	0.336***	1.000								
Tma	0.351***	0.337***	1.000							1.04
RD	0.366***	0.310***	0.287***	1.000						1.10
Lev	−0.541***	−0.064**	−0.209***	−0.282***	1.000					1.14
Size	0.274***	0.596***	0.204***	0.124***	0.073***	1.000				1.15
Gro	0.317***	0.268***	0.391***	0.199***	−0.288***	0.116***	1.000			1.02
AT	0.339***	0.353***	0.227***	−0.098***	0.019	0.317***	0.125***	1.000		1.10
SA	−0.119***	−0.346***	−0.328***	−0.121***	−0.044*	−0.399***	−0.306***	−0.246***	1.000	1.29

*, **, *** indicate significant at the 10 percent, 5 percent, and 1 percent levels, respectively, with standard errors in parentheses.

### 4.3 Benchmark regression results

A fixed effects model is used to test the impact of technology mergers and acquisitions on pharmaceutical firms’ performance, and the regression results of model (1) are shown in Table 4. Column (1)–Column (4) regressions are all controlled for individual and time effects. Columns (1) and (2) are the effects of technology mergers and acquisitions on the financial performance of enterprises. In column (1), the regression coefficient of technology mergers and acquisitions (Tma) on Roa is 0.0552, which is significant at the 1% statistical level. In column (2), after adding control variables, the coefficient is 0.0149 and still significant at the 1% level, which means that technology mergers and acquisitions can significantly contribute to the enhancement of the financial performance of pharmaceutical enterprises. This implies that technology mergers and acquisitions can significantly promote the financial performance of pharmaceutical companies. Columns (3) and (4) show the effect of technology M&A on innovation performance. Column (3) does not include control variables, and column (4) adds control variables. The regression coefficients of technology M&A are 0.3885 and 0.0881, respectively, which are significant at the 1 percent confidence level, indicating that technology M&A can also enhance the innovation performance of pharmaceutical enterprises; this validates H1. Based on theoretical analysis, enterprises that acquire technology resources for M&As pay more attention to integrating technology and knowledge to quickly absorb the acquired enterprise’s technical knowledge. It can better enhance corporate performance by expanding the scale of their basic knowledge. In addition, by comparing the regression findings in columns (2) and (4), it is evident that technology M&A has a more pronounced impact on pharmaceutical firms’ innovation performance than its effect on financial performance. It could be because technology mergers and acquisitions may prompt firms to adjust their patent policies. Companies can evaluate and modify their patent portfolios based on market demand and technological advancements throughout the merger and acquisition process. As a result, companies may choose to augment their patent applications to align with the changing market conditions and competitive forces.

**Table 4:** Benchmark regression results.

Variable	Roa	Roa	Invia	Invia
	(1)	(2)	(3)	(4)
Tma	0.0552***	0.0149***	0.3885***	0.0881***
	(0.0035)	(0.0042)	(0.0224)	(0.0254)
Lev		−0.1137***		−0.3316**
		(0.0261)		(0.1367)
Size		0.0366***		0.4950***
		(0.0120)		(0.0931)
Gro		0.0417***		0.1479**
		(0.0099)		(0.0625)
AT		0.0813***		0.1832*
		(0.0197)		(0.1081)
SA		0.0107		−0.3995***
		(0.0180)		(0.0874)
_cons	0.0433***	−0.2626**	0.6950***	−5.4888***
	(0.0036)	(0.1115)	(0.0337)	(0.8510)
Year	Yes	Yes	Yes	Yes
Id	Yes	Yes	Yes	Yes
adj.*R*^2^	0.2365	0.5352	0.2483	0.5688
*N*	1,418	1,418	1,418	1,418

*, **, *** indicate significant at the 10 percent, 5 percent, and 1 percent levels, respectively, with standard errors in parentheses.

### 4.4 Mediating effects of R&D inputs

Table 5 shows the regression results of research and development investment (RD) as a mediating variable. According to the three-step method of mediating effect, columns (1) and (4) are the results of the benchmark regression of Tma on firm performance, consistent with the above results. Column (2) shows the effect of technology mergers and acquisitions on RD, and the regression coefficient of RD is 1.134 and is significantly positive at the 1% level. It indicates that technology mergers and acquisitions positively impact enterprise R&D investment, and enterprise R&D investment is significantly improved after the enterprise carries out technology mergers and acquisitions. Firms obtain new R&D capabilities and technological knowledge through technology mergers and acquisitions. To fully use these new R&D capabilities, firms increase their R&D investment to develop more innovative products and technologies. Columns (3) and (5) show the effects of technology M&A on firms’ financial performance and innovation performance after adding the mediating variable of RD, respectively. The coefficients of Tma on Roa are 0.00999, which is significantly positive at a 10% statistical level, respectively. The coefficient of Tma on Invia is 0.0673, which is significantly positive at a 5% statistical level. This indicates that R&D investment mediates the effect of technology mergers and acquisitions on enterprise performance, thus verifying the above H2. After a technological merger and acquisition, boosting R&D investment can expedite the company’s integration and assimilation of external technology resources and core knowledge skills. Enterprises can enhance their level of technological innovation by bolstering internal research and development efforts and aggressively leveraging them. Simultaneously, the surge in research and development spending resulting from technological M&A aids firms in effectively adapting to shifts in market demand, thereby enhancing their performance.

**Table 5:** Regression results mediated by R&D investment.

Variable	Roa	RD	Roa	Invia	Invia
	(1)	(2)	(3)	(4)	(5)
RD			0.00434***		0.0183**
			(5.63)		(3.31)
Tma	0.0149***	1.134***	0.00999*	0.0881***	0.0673**
	(3.52)	(4.89)	(2.37)	(3.47)	(2.64)
Lev	−0.114***	−3.022**	−0.101***	−0.332*	−0.276*
	(−4.36)	(−2.71)	(−4.13)	(−2.43)	(−2.06)
Size	0.0366**	1.392*	0.0306**	0.495***	0.470***
	(3.05)	(2.27)	(2.68)	(5.32)	(5.19)
Gro	0.0417***	0.480	0.0396***	0.148*	0.139*
	(4.20)	(1.04)	(4.20)	(2.37)	(2.23)
AT	0.0813***	0.160	0.0806***	0.183	0.180
	(4.13)	(0.18)	(4.34)	(1.70)	(1.70)
SA	0.0107	−2.597**	0.0219	−0.399***	−0.352***
	(0.59)	(−2.74)	(1.31)	(−4.57)	(−4.11)
_cons	−0.263*	−17.95**	−0.185	−5.489***	−5.160***
	(−2.35)	(−3.31)	(−1.74)	(−6.45)	(−6.27)
Year	Yes	Yes	Yes	Yes	Yes
Id	Yes	Yes	Yes	Yes	Yes
N	1,418	1,418	1,418	1,418	1,418
R2	0.540	0.353	0.569	0.574	0.584
adj. R2	0.535	0.345	0.564	0.569	0.579

*, **, *** indicate significant at the 10 percent, 5 percent, and 1 percent levels, respectively, with standard errors in parentheses.

In addition, the model passed the Sobel test and Bootstrap test (drawing self-help samples 1,000 times), and the test results are shown in Table 6. When the explanatory variable is Roa, the direct effect coefficient is 0.009992, and the indirect effect coefficient is 0.00492, which is significantly positive at 1%. When the explanatory variable is Invia, the direct effect coefficient is 0.06782, and the indirect effect coefficient is 0.20802, both of which are significant at 1%. It indicates that research and development investment (RD) as a mediating variable promotes the positive effect of technology mergers and acquisitions on firms’ financial performance, further validating H2.

**Table 6:** Mediating effect model Sobel and Bootstrap test.

Sobel-Goodman Mediation Tests		
Variable	Roa	Invia
Indirect effect	0.00492*** (*z* = 5.71257)	0.20802*** (*z* = 4.47182)
Direct effect	0.009992*** (*z* = 3.761)	0.06782*** (*z* = 3.68837)
Total effect	0.014912*** (*z* = 5.54894)	0.088082*** (*z* = 4.87)
Ratio of indirect to direct effect	0.49241216	0.30919048

Bootstrap Tests		
Variable	Roa	Invia
bs_1	0.0076012*** (*z* = 6.05)	0.0612099*** (*z* = 5.43)
Lower limit	0.0051394	0.0391322
Upper limit	0.0100631	0.0832876
bs_2	0.013359*** (*z* = 3.54)	0.1045136*** (*z* = 3.91)
Lower limit	0.0059613	0.0520775
Upper limit	0.0207506	0.1569497

*, **, *** indicate significant at the 10 percent, 5 percent, and 1 percent levels, respectively, with standard errors in parentheses.

### 4.5 Robustness tests

#### 4.5.1 Replacement of variable indicators

Referring to the study of Wang and Huang (46), this paper replaces the dummy variable of whether the explanatory variable is mergers and acquisitions (Tma) with the ratio of the sum of the amount of all technology merger and acquisition deals initiated by listed companies in the year to the total assets (Ta) to conduct the regression again, and the results are shown in columns (1) and (2) of Table 7. The coefficient of Roa is 0.1517, the coefficient of Invia is 0.2078, and the regression coefficients of firm performance are all significantly positive at the 1% level. This indicates that technology mergers and acquisitions positively impact firm performance, and the regression results are consistent with the benchmark regression results. The explanatory variable return on assets (Roa) is replaced by return on equity (Roe), and the regression results are shown in column (3) of Table 7. The coefficient of Roe is 0.0159, which is significantly positive at the 10 percent level. The regression results are consistent with those of the benchmark regression results, which indicates that this paper’s conclusion on the promotional effect of technology mergers and acquisitions on corporate performance is robust.

**Table 7:** Robustness test.

Variable	Substitution of variable indicators			Sample reconstruction		Replacement model	
	Roa	Invia	Roe	Roa	Invia	Roa	Invia
	(1)	(2)	(3)	(4)	(5)	(6)	(7)
Ta	0.1517***	0.2078***					
	(0.0358)	(0.3027)					
Tma			0.0159*	0.0244***	0.3121***	0.0153***	0.0874***
			(0.0095)	(0.0043)	(0.0319)	(0.0043)	(0.0256)
Lev	−0.0891***	−0.1925***	−0.2416***	−0.1560***	0.1703**	−0.1175***	−0.3360**
	(0.0147)	(0.1344)	(0.0843)	(0.0099)	(0.0733)	(0.0269)	(0.1380)
Size	0.0413**	0.3725***	0.1665***	0.0525***	0.5377***	0.0309**	0.4817***
	(0.0168)	(0.1104)	(0.0453)	(0.0045)	(0.0331)	(0.0122)	(0.0940)
Gro	0.0203***	0.0004**	0.0481***	0.0116***	−0.0055	0.0444***	0.1555**
	(0.0048)	(0.0454)	(0.0179)	(0.0038)	(0.0280)	(0.0104)	(0.0617)
AT	0.0889***	0.0659**	0.2586***	0.0800***	0.1422***	0.0805***	0.1956*
	(0.0223)	(0.1169)	(0.0710)	(0.0071)	(0.0525)	(0.0210)	(0.1102)
SA	−0.1281***	−0.2688***	0.3117***	0.0768***	0.1372***	0.0134	−0.3927***
	(0.0141)	(0.0839)	(0.0592)	(0.0071)	(0.0522)	(0.0180)	(0.0857)
Year	Yes	Yes	Yes	Yes	Yes	Yes	Yes
Id	Yes	Yes	Yes	Yes	Yes	Yes	Yes
_cons	−0.0002	−2.1807**	−0.3786	−0.1424***	−3.9005***	-0.1984***	-5.3091***
	(0.1425)	(0.8620)	(0.5275)	(0.0461)	(0.3416)	(0.1155)	(0.8503)
*N*	1,418	1,418	1,418	1,144	1,144	1,418	1,418
adj.*R*^2^	0.5125	0.2097	0.2122	0.4047	0.2914	0.5238	0.5674

*, **, *** indicate significant at the 10 percent, 5 percent, and 1 percent levels, respectively, with standard errors in parentheses.

#### 4.5.2 Reconstructing the sample

Referring to the empirical research method of Li and Yang (50), this paper transforms the unbalanced panel data into balanced panel data for regression to ensure sample integrity. The regression results are shown in columns (4) and (5) of Table 7. The coefficient of Roa is 0.0244, the coefficient of Invia is 0.3121, and the coefficient of technology M&A on firm performance is still significantly positive and significant at the 1% level, indicating that the benchmark regression results are robust and reliable.

#### 4.5.3 Replacement of measurement model

To avoid the influence of problems such as autocorrelation and heteroskedasticity, this paper refers to the study of Zhang et al. (51), adjusts the heteroskedasticity and clustering of the standard errors, and shows the results in Table 7. From the regression results in Columns (6) and (7), it can be seen that, after the adjustments to the standard errors, the promotional effect of technology M&As on firm performance is still significant, which once again verifies the robustness of the conclusions of the paper’s study.

### 4.6 Endogeneity test

The endogeneity problem is usually a 3-pronged problem of omitted variables, bi-directional causality, and measurement error in the variables. To eliminate the possibility of endogeneity problems, this paper adopts instrumental variables and the dynamic panel system generalized moment estimation (SYS-GMM) method for testing. This paper refers to the study of Li et al. (52) and adopts the explanatory variables (Tma) lagged term (L.Tma) as an instrumental variable, which can keep the obvious correlation between it and the explanatory variables, and also avoid the problem of weak instrumental variables. In addition, the current period’s disturbance terms cannot affect these lagged indicators. Therefore, the instrumental variables are selected to lag the lagged terms of the explanatory variables, which can satisfy the constraints of correlation and homogeneity.

Table 8 shows the results of the instrumental variable test. After controlling for possible endogeneity issues by choosing this instrumental variable, the Roa and Invia coefficients are still positive, the level of significance remains unchanged, and technological M&A still present significant positive incentives for firm performance. The results of this test once again maintain the findings of the previous study, indicating that the results are robust and credible.

**Table 8:** Endogeneity test (instrumental variable approach).

Variable	Roa	Tma	Roa	Invia	Tma	Invia
	(1)	(2)	(3)	(4)	(5)	(6)
Tma	0.0240***			0.3158***		
	(−0.004)			(−0.0267)		
L.Tma		0.4326***	0.0362***		0.4326***	0.2866***
		(−0.0368)	(−0.0094)		(−0.0368)	(−0.0716)
Lev	−0.1521***	0.1401**	−0.1557***	0.2034***	0.1401**	0.2195***
	(−0.0138)	(−0.0607)	(−0.0152)	(−0.0689)	(−0.0607)	(−0.0731)
Size	0.0491***	0.0034	0.0522***	0.5050***	0.0034	0.5077***
	(−0.0051)	(−0.0297)	(−0.0055)	(−0.0299)	(−0.0297)	(−0.0312)
Gro	0.0123**	−0.034	0.0118*	0.0019**	−0.034	−0.0048*
	(−0.0077)	(−0.0222)	(−0.0081)	(−0.0222)	(−0.0222)	(−0.0237)
AT	0.0800***	0.0766	0.0791***	0.1481***	0.0766	0.1509***
	(−0.0071)	(−0.0489)	(−0.008)	(−0.0532)	(−0.0489)	(−0.0574)
SA	0.0829***	0.2947***	0.0818***	0.1997***	0.2947***	0.2096***
	(−0.0082)	(−0.0496)	(−0.0092)	(−0.0523)	(−0.0496)	(−0.0597)
_cons	−0.0953**	1.1398***	−0.1303***	−3.3918***	1.1398***	−3.3807***
	(−0.0435)	(−0.2968)	(−0.0494)	(−0.3152)	(−0.2968)	(−0.342)
*N*	1,418	1,040	1,040	1,418	1,040	1,040
adj. *R*^2^	0.3989	0.2448	0.3986	0.2677	0.2448	0.27

*, **, *** indicate significant at the 10 percent, 5 percent, and 1 percent levels, respectively, with standard errors in parentheses.

In addition, this paper incorporates the lagged one-period of the explanatory variables into the regression model to further address possible endogeneity issues through the SYS-GMM approach. In the SYS-GMM estimation, we consider the lagged one period of the explanatory variables and technology mergers and acquisitions as endogenous variables and use the lagged terms of the explanatory variables as instrumental variables. Roodman (53) emphasizes that the HansenTest is more robust than the SarganTest regarding heteroskedasticity problems in the model. Hence, this paper reports the results of the HansenTest. The results of the endogeneity test are shown in Table 9, where we find that the regression coefficients of the explanatory variables return on total assets (Roa) lagged one period is 0.1442, which is significantly positive at the 10% level. The coefficient of the number of invention patent applications (Invia) is 0.2269, which is significantly positive at the 5% level. This indicates that there is an inertia in the firms’ technology M&A decisions and that the outcome of the decision in the previous period significantly affects the technology M&A decisions in the next period. The regression coefficients of the explanatory variable technology mergers and acquisitions (Tma) are 0.0315 and 0.3621, respectively, which are still significantly positive at the 1 percent confidence level. To enhance the reliability of the SYS-GMM estimation results, the rationality of the model setup and the validity of the instrumental variable selection are examined in this paper, respectively. Among them, the test results of AR(2) for second-order serial correlation show that the original hypothesis cannot be rejected, indicating that there is no second-order serial correlation in the residual terms of the dynamic panel. Also, the results of the HansenTest for the test of whether there is over-identification of instrumental variables indicate that the instrumental variables used in the model are appropriate.

**Table 9:** Endogeneity test (SYS-GMM).

Variable	SYS-GMM	
	Roa	Invia
	(1)	(2)
LRoa	0.1442*	
	(0.0760)	
L.Invia		0.2269**
		(0.0967)
Tma	0.0315***	0.3621***
	(0.0109)	(0.0898)
Lev	−0.1072**	−0.0315
	(0.0490)	(0.3293)
Size	0.0979***	0.2351
	(0.0252)	(0.1991)
Gro	0.0058	−0.3752
	(0.0487)	(0.3811)
AT	0.0721	−0.0564
	(0.0689)	(0.4101)
SA	0.0671	−0.4982
	(0.0625)	(0.5619)
Year	Yes	Yes
Id	Yes	Yes
*F*-value	22.71	13.18
AR(1)	0.012	0.091
AR(1)	0.401	0.601
Hansen test	0.256	0.069
*N*	715	715

*, **, *** indicate significant at the 10 percent, 5 percent, and 1 percent levels, respectively, with standard errors in parentheses.

## 5 Heterogeneity analysis

### 5.1 Heterogeneity in the nature of corporate equity

Organizational structures and management styles may vary between firms due to equity variances. This paper examines the nature of equity based on information from the actual controller of listed companies in the Cathay Pacific database. The sample is divided into state-owned enterprises and non-state-owned enterprises, including private, foreign, and other types of enterprises. The data is then analyzed using a fixed effects model for regression to determine the difference in the impact of technology mergers and acquisitions on enterprise performance.

Table 10 shows the results of the equity heterogeneity test. Overall, for both state-owned and non-state-owned enterprises, technology mergers and acquisitions significantly impact enterprise performance, which verifies the robustness of the benchmark regression results. Specifically, compared to state-owned firms, technological M&As have a higher positive impact on the performance of non-state-owned enterprises. According to the theoretical study, this could be because state-owned firms have a more comprehensive range of reasons for engaging in technological mergers and acquisitions, and they prioritize objectives other than innovation performance. Non-state-owned enterprises typically encounter heightened market competition and prioritize assimilating technology and knowledge following technology mergers and acquisitions. It enables the enterprises to swiftly incorporate the acquired company’s technological expertise, enhancing benefits. In addition, non-state-owned firms exhibit greater adaptability and prowess in innovation than state-owned counterparts. State-owned firms could face additional legislative limitations and regulations, which could impede their ability to innovate. Technology M&As can offer non-state-owned companies access to fresh technology and expertise, enabling them to enhance efficiency, save expenses, and innovate new products.

**Table 10:** Equity heterogeneity regression results.

Variable	Heterogeneity in the nature of corporate equity			
	Non-state-owned enterprises		State-owned enterprises	
	Roa	Invia	Roa	Invia
Tma	0.0187***	0.3065***	0.0090**	0.2894***
	(0.0036)	(0.0212)	(0.0038)	(0.0469)
Lev	−0.0958***	−0.0282	−0.0774***	−0.1530
	(0.0134)	(0.1208)	(0.0237)	(0.2825)
Size	0.0630***	0.3055***	0.0346	0.5357**
	(0.0150)	(0.0925)	(0.0294)	(0.2289)
Gro	0.0145***	−0.0384	0.0173**	−0.0330
	(0.0049)	(0.0383)	(0.0073)	(0.0604)
AT	0.1054***	−0.1596	0.0877**	−0.1238
	(0.0190)	(0.1016)	(0.0344)	(0.2027)
SA	0.1300***	0.1793**	0.0980***	−0.1680
	(0.0165)	(0.0773)	(0.0207)	(0.1062)
Year	Yes	Yes	Yes	Yes
Id	Yes	Yes	Yes	Yes
_cons	−0.0638	−1.3573	0.0643	−4.8265*
	(0.1606)	(0.9344)	(0.2895)	(2.3975)
*N*	1,033	1,033	385	385
adj.*R*^2^	0.5510	0.2316	0.4437	0.2056

*, **, *** indicate significant at the 10 percent, 5 percent, and 1 percent levels, respectively, with standard errors in parentheses.

### 5.2 Firm size heterogeneity

The size of a firm is a crucial aspect that affects how technology mergers and acquisitions impact the performance of the firm. This paper categorizes the enterprise’s total assets at the end of the period into two groups: the first group includes large-scale enterprises with total assets in the first three quartiles of the size distribution, while the second group includes small and medium-sized enterprises with the remaining total assets.

Table 11 shows the regression outcomes for various company sizes, indicating that technological M&As have a substantial impact on the performance of both large-scale and small-and medium-sized pharmaceutical companies. Specifically, technology M&As have a more significant impact on enhancing innovation performance in small and medium-sized companies than in large-scale organizations. This is because small-scale enterprises typically possess a more uniform business plan, product assortment, and a very uncomplicated management structure. They can respond and take action with incredible speed and adaptability when confronted with technology mergers and acquisitions. Furthermore, they are eager to capitalize on the opportunity presented by technology M&As to enhance technological innovation. However, the inflexibility inherent in the hierarchical structure of large-scale corporations, as opposed to small-scale enterprises, diminishes the motivation for technical innovation. In addition, large corporations own more advanced infrastructure, typically have more sophisticated innovation frameworks, and may require less emphasis on innovation. Technology mergers and acquisitions are more effective in fostering the growth of large-scale enterprises in terms of financial performance. Based on the theoretical analysis, this may be attributed to variations in the knowledge resources, the ability to integrate and absorb new information, and the capability to finance research and development among firms of varying sizes. Technology M&As frequently necessitate a significant financial commitment to support ongoing research and development spending and the integration of resources. Major corporations consistently secure additional research and development funding following a technology merger and acquisition, whereas smaller companies may encounter financial limitations shortly after completing a technology merger and acquisition. Small-sized enterprises may lack the financial resources to cover the increased expenses of mergers and acquisitions and the consequent investment in research and development. Furthermore, small-scale firms often have limited research and development capabilities and struggle to effectively integrate resources, which contrasts with the more robust capabilities of large-scale enterprises. This disparity can result in inadequate technical integration and innovation following a merger or acquisition, ultimately impacting the financial performance of the enterprises.

**Table 11:** Regression results of size heterogeneity.

Variable	Firm size heterogeneity			
	Large-scale firms		Small-and medium-sized firms	
	Roa	Invia	Roa	Invia
Tma	0.0191***	0.2881***	0.0165***	0.3272***
	(0.0055)	(0.0377)	(0.0034)	(0.0227)
Lev	−0.0948***	−0.3391	−0.0989***	−0.0534
	(0.0320)	(0.2909)	(0.0125)	(0.1297)
Size	0.0069	0.6379**	0.0636***	0.3521***
	(0.0215)	(0.2551)	(0.0141)	(0.1108)
Gro	−0.0002	−0.1126*	0.0243***	−0.0011
	(0.0080)	(0.0565)	(0.0049)	(0.0376)
AT	0.0708***	0.0561	0.0881***	−0.1863*
	(0.0227)	(0.1785)	(0.0162)	(0.1006)
SA	0.1311***	0.0303	0.1208***	0.1385*
	(0.0206)	(0.0934)	(0.0159)	(0.0829)
Year	Yes	Yes	Yes	Yes
Id	Yes	Yes	Yes	Yes
_cons	0.4882**	−5.0416*	−0.0957	−1.9921*
	(0.2278)	(2.7039)	(0.1412)	(1.1511)
*N*	426	426	992	992
adj.*R*^2^	0.4689	0.2838	0.5446	0.2188

*, **, *** indicate significant at the 10 percent, 5 percent, and 1 percent levels, respectively, with standard errors in parentheses.

### 5.3 Regional heterogeneity

The geographical distribution of pharmaceutical firms in China is divided into several regions. Therefore, we categorize the listed pharmaceutical companies in our sample into three groups: East, Central, and West. The sample consists of 648 pharmaceutical enterprises in the East area, 279 in the Central region, and 208 in the West region. Subsequently, we assess the influence of technological mergers and acquisitions on the corporate performance of pharmaceutical firms in these three geographical areas.

Table 12 shows the outcomes of the examination of regional heterogeneity. The regression analysis indicates that technological M&As have a substantial impact on the performance of pharmaceutical companies in the central, eastern, and western areas. More precisely, the impact of technological M&As on the performance of companies is more noticeable in the eastern region than in the central and western areas. The reason for this could be attributed to the fact that the east part of China is primarily located along the coastline, characterized by a flat topography, efficient transportation infrastructure, and the presence of numerous prominent domestic pharmaceutical companies. Consequently, this region has become a magnet for drawing many highly skilled professionals (54). Pharmaceutical businesses in the eastern area are increasing their collaboration and engagement with major multinational pharmaceutical corporations, displaying greater agility in responding to industry dynamics and adopting a more proactive strategy toward mergers and acquisitions. Relatively speaking, China’s central and western areas began to engage with the international community later, and their infrastructure development is comparatively less advanced. The natural environmental conditions in this area are subpar, and its economic progress is sluggish, characterized by a scarcity of technology, skilled individuals, and financial resources. In addition, according to the theoretical aspect of the preceding analysis, the central and western regions exhibit a generally low level of technology, resulting in limited capacity for digestion and absorption and a comparatively poor technological knowledge base. Consequently, it is difficult to bring about major technological breakthroughs for remote regions because they may struggle to completely comprehend and integrate the company’s technological advancements following the merger and acquisition.

**Table 12:** Geographical heterogeneity regression results.

Variable	Roa			Invia		
	East	Middle	West	East	Middle	West
	(1)	(2)	(3)	(4)	(5)	(6)
Tma	0.0293***	0.0158**	0.0106*	0.3558***	0.3335***	0.2922***
	(−0.0099)	(−0.0071)	(−0.0057)	(−0.0549)	(−0.041)	(−0.0347)
Lev	−0.1515***	−0.0604	−0.1401***	−0.0285	−0.0734*	−0.0969*
	(−0.0338)	(−0.0412)	(−0.0398)	(−0.3961)	(−0.2633)	(−0.184)
Size	−0.0129	0.0962***	0.0466	0.154	0.5976***	0.3658***
	(−0.0217)	(−0.0332)	(−0.0322)	(−0.1995)	(−0.1991)	(−0.1277)
Gro	0.0314***	0.0078	0.0108	−0.0324	−0.0841	0.0018
	(−0.0094)	(−0.0085)	(−0.0093)	(−0.0692)	(−0.0645)	(−0.0165)
AT	−0.0039	0.1356***	0.0991***	−0.3424**	0.3975	−0.1861
	(−0.0391)	(−0.0374)	(−0.0335)	(−0.1568)	(−0.2755)	(−0.1516)
SA	0.1046**	0.0935***	0.1465***	0.2786*	0.2042	−0.0107
	(−0.0401)	(−0.0289)	(−0.0186)	(−0.1403)	(−0.1538)	(−0.1125)
Year	Yes	Yes	Yes	Yes	Yes	Yes
Id	Yes	Yes	Yes	Yes	Yes	Yes
_cons	0.6137**	−0.542	0.1544	0.5269	−4.3674**	−2.4935*
	−0.238	−0.3599	−0.326	−1.9162	−1.9365	−1.4495
*N*	648	279	208	648	279	208
adj. *R*^2^	0.2978	0.4563	0.4478	0.2182	0.2052	0.2052

*, **, *** indicate significant at the 10 percent, 5 percent, and 1 percent levels, respectively, with standard errors in parentheses.

## 6 Conclusion and recommendations

### 6.1 Conclusion

The macro background of China’s economic structural transformation requires enterprises to have more robust technological innovation capabilities. Technology mergers and acquisitions (M&A) are ineffective strategies to occupy a favorable market position. This paper empirically analyses the impact of technology mergers and acquisitions (M&A) on enterprise performance with the sample data of Chinese pharmaceutical-listed enterprises from 2012 to 2022. The conclusions are as follows: (i) Benchmark regression results show that technology mergers and acquisitions (M&A) have a significant positive impact on the performance of pharmaceutical enterprises. (ii) Based on the mediation effect model, it is found that there is a mediation effect of R&D investment in the impact of technology M&As on enterprise performance. (iii) Through subgroup regression, the effect of technology M&A on pharmaceutical firm performance is heterogeneous regarding the nature of equity, firm size, and geographic region. Regarding different equity natures, technology M&A has a more vital role in promoting the performance of non-state-owned enterprises than state-owned enterprises. Regarding firm size, technology M&As have a more substantial effect on the innovation performance of small and medium-sized enterprises than large firms and a stronger effect on the financial performance of large firms than small and medium-sized enterprises. Regarding different regions, technology M&As are more effective in promoting the performance of pharmaceutical enterprises in the eastern region.

### 6.2 Recommendations

Based on the above theoretical and empirical results, combined with the status quo of technology M&A activities and R&D investment of China’s listed pharmaceutical companies, this paper proposes suggestions from both the government and enterprise levels.

At the governmental level, it is imperative for the government to enhance the focus on technology M&As to foster enterprise innovation. Additionally, the government should provide guidance and incentives to pharmaceutical companies to improve their performance using technology M&A. Acknowledging the diversity of technology M&As in enhancing innovation performance across organizations with varying ownership structures, sizes, and geographical locations is essential. When implementing the policy, it is crucial to prioritize efficiency and balance to expedite achieving the goal of supporting technological innovation and enterprise development. It will ensure that technology mergers and acquisitions activities provide the most favorable outcomes. Specifically, the government should consider the different behavior of enterprises with different property rights in the face of technology mergers and acquisitions. The government should provide state-owned firms a permissive environment for technology mergers and acquisitions. It is recommended that the current obstacles preventing state-owned firms from participating in technology M&A be removed by streamlining the administrative license and approval processes. Enterprise innovation necessitates substantial cash; the government can implement policies such as providing financial subsidies and tax incentives. It can alleviate the difficulties encountered by enterprises in the process of technology mergers and acquisitions by strengthening the government’s financial support. Secondly, in the face of the differences between pharmaceutical production enterprises of different sizes. The Government should provide guidance and support to foster the growth of innovative and competitive small and medium-sized firms in the industry. It will modify its policy’s flexibility to enhance the diverse market demand by setting aside a specific market share for qualified businesses. The Government also stimulates large-scale enterprises to merge and acquire overseas high-tech enterprises and integrate domestic resources by establishing special funds and technical support measures to promote competitiveness and innovation in the pharmaceutical market. Finally, the government should fully consider the actual differences in the situation of pharmaceutical production enterprises in the eastern, central, and western regions. It should strengthen support for the M&A and innovation system in those areas and facilitate technical mergers and acquisitions by bringing in talent and bolstering infrastructure to boost innovation in the central and western regions. Simultaneously, the government should establish a cross-regional collaboration platform for enterprises in the eastern, central, and western areas to facilitate the sharing of resources and foster collaborative innovation. This platform encourages the involvement of enterprises from the central and western regions in merger and acquisition activities to enhance their capability and success rate in M&A endeavors.

At the company level, it is crucial for companies to recognize the significance of innovation to differentiate themselves in the competitive market. Technology M&A and investment in R&D are successful strategies for organizations to obtain innovative resources and improve their ability to innovate technologically in response to changes in their business models. State-owned enterprises should leverage their resources and fully capitalize on their strengths. They should demonstrate the bravery to expand internationally and actively engage in mergers and acquisitions to foster innovation. In addition, enterprises must comprehend cutting-edge market trends, consistently acquire and assimilate sophisticated technological expertise, and augment their consciousness of innovation and research and development capabilities. Non-state-owned enterprises must align with national strategies, persist in exploring and innovating, and enhance their investment in research and development. They should focus on innovation and quality and offer a wide range of items to cater to consumers’ diverse needs to gain a competitive edge in the market. It will help them avoid competing with similar products. Large enterprises should leverage their extensive technological expertise and consistently innovate by building upon their existing technology to improve the level of R&D and enhance the core competitiveness of firms in the industry. Small and medium-sized enterprises should evaluate their capacity for growth and determine if they can obtain external technological resources by engaging in technology mergers and acquisitions. It is essential to thoroughly assess the potential risks and advantages of technology M&As and develop a comprehensive M&A strategy that effectively incorporates technology and accelerates technology upgrading smoothly. Also, enhancing the accumulation of financial resources and human capital is imperative. They should exercise stringent control over the allocation of research and development funding, attract exceptionally skilled individuals to increase the scope of corporate knowledge, and strengthen the fundamental competitiveness of firms. In addition, it is crucial to be mindful of market trends and fully comprehend the cutting-edge advancements within the sector. Pharmaceutical enterprises in the central and western regions should enhance their collaboration with external firms that possess robust research and development capabilities, as well as universities and research institutes. By combining industry, academia, and research, they can enhance the existing technology level, breaking through the bottleneck and improving the success rate of technology M&A. At the same time, enterprises should consider improving the welfare treatment of talents and strengthening the training of talents to ensure the long-term development of enterprises.

## 7 Shortcomings and prospects

This study still has many shortcomings, which may also be worth further exploration. First, this paper only studied the pharmaceutical manufacturing industry in China. Although pharmaceutical manufacturing enterprises are typical representatives of the real economy and high-tech economy, the research object is still limited and can expand the scope of the study to other industries in other countries in the future. Second, due to the availability of data, this paper only considered listed pharmaceutical enterprises, and the phenomenon of technological mergers and acquisitions also exists in non-listed pharmaceutical enterprises. Future research can turn to unlisted companies to fully understand the relationship between technology M&A and firm performance. Third, this paper only analyzes the correlation from the relationship between technology M&A, R&D investment, and firm performance, but in practice, there may be other unobserved factors (such as market competition, managerial decision-making, etc.) affecting firm performance, so it is necessary to explore the correlation analysis more deeply in the future.

## Data availability statement

The datasets presented in this study can be found in online repositories. The names of the repository/repositories and accession number(s) can be found in the article/supplementary material.

## Author contributions

JY: Conceptualization, Data curation, Formal analysis, Investigation, Methodology, Project administration, Validation, Writing – original draft. JL: Methodology, Validation, Writing – review & editing. SW: Supervision, Writing – review & editing. YC: Supervision, Writing – review & editing.

## Footnotes

^1^ http://www.cninfo.com/
